# An Improved Model for the hTERT Promoter Quadruplex

**DOI:** 10.1371/journal.pone.0115580

**Published:** 2014-12-19

**Authors:** Jonathan B. Chaires, John O. Trent, Robert D. Gray, William L. Dean, Robert Buscaglia, Shelia D. Thomas, Donald M. Miller

**Affiliations:** James Graham Brown Cancer Center, Department of Medicine, University of Louisville, Louisville, Kentucky, United States of America; Imperial College London, United Kingdom

## Abstract

Mutations occur at four specific sites in the hTERT promoter in >75% of glioblastomas and melanomas, but the mechanism by which the mutations affect gene expression remains unexplained. We report biophysical computational studies that show that the hTERT promoter sequence forms a novel G-quadruplex structure consisting of three contiguous, stacked parallel quadruplexes. The reported hTERT mutations map to the central quadruplex within this structure, and lead to an alteration of its hydrodynamic properties and stability.

## Introduction

Over the past decade, genomic DNA sequencing efforts have revealed the broad mutational landscapes of common human cancers [1]. Despite these advances, mutations in the promoters of cancer genes have not been documented as a common cause of gene dysregulation in cancer. Using whole genome sequencing data, several groups have recently shown that mutations occur at four specific sites in the hTERT promoter [2]–[4] in >75% of glioblastomas and melanomas. Additionally, the cooperation of BRAF and hTERT mutations was recently reported in aggressive thyroid cancer [5]. Some of these mutations involve cytosine to thymine transitions suggesting that, in the case of melanoma, they may be UV induced. The guanine to adenine mutations create a new binding site for the E-twenty six (ETS) transcription factor, which has been hypothesized to be the mechanism of increased hTERT expression [2], [3].

We noticed that these mutations all occur in a G-rich region of the hTERT promoter which has previously been shown to form quadruplex DNA [6]. We speculated that the occurrence of these mutations could destabilize, or alter the recognition of, quadruplexes formed by this sequence. This would be expected to abrogate the negative effect of quadruplex formation on the transcriptional activity of the hTERT promoter, allowing increased hTERT expression.

Quadruplex DNA is a four-stranded structure that is stabilized by G-quartets (four guanines which interact via Hoogsteen hydrogen bonding to form a planar tetrad ring [7], [8]). There are more than 370,000 putative quadruplex-forming sequences (QFS) in the human genome, which are disproportionately represented in the promoters of growth regulatory genes [9], [10]. Roughly half of human promoters contain QFS. These sequences can form either intramolecular or intermolecular Hoogsteen hydrogen bonds stabilizing their secondary and tertiary structure [8], [11]. Recent work has provided clear evidence that quadruplex structures exist in both RNA and DNA *in vivo* eukaryotes [12]–[14]. The QFS in the c-myc [15], c-Myb [16], K-Ras [17], Bcl-2 [18], [19], retinoblastoma [20] and HIF1α [21] gene promoters are all in important regulatory regions. In the instance of c-myc and hTERT, the molecular function has been characterized and there is clear evidence that quadruplex formation inhibits gene expression, presumably by silencing transcription [6], [22]. Ironically, quadruplex-forming sequences also occur in telomeric DNA [23], [24] helping provide protection for chromosomal ends.

**Figure 1:**
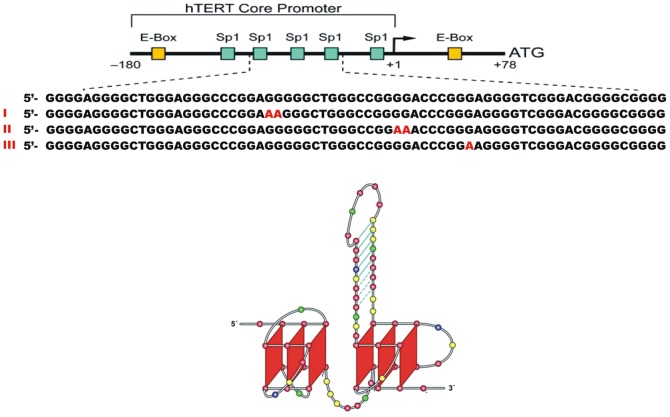
The hTERT promoter sequence and proposed structure. *Top*: Sequence of the hTERT core promoter with the positions of mutations observed in melanomas shown in red. *Bottom*: Previously proposed structure of the hTERT core promoter (5). Adapted with permission from (5).

The locations of the reported hTERT promoter mutations are shown in Fig. 1, along with the structure of the hTERT promoter proposed by Palumbo *et. al* [6]. In that structure the reported G to A transitions would be somewhat oddly placed with one in the hairpin loop, one in the duplex stem and one in the antiparallel quadruplex. The effects of these transitions on the stability and function of the proposed structure are by no means clear. We describe here a new detailed model for the hTERT quadruplex structure completely consistent with biophysical data. In the new model, mutations are localized within a single central G-quadruplex. The mutations may alter the stability and molecular recognition of the hTERT quadruplex.

## Materials and Methods

### Oligodeoxynucleotides

Names, sequences and absorption coefficients of the oligonucleotides used in this study are given in Table 1. hTERT (obtained from Oligos Etc.) was dissolved in water at a concentration of 650 µM. 1XAV was from IDT, Coralville, IA. The lyophilized, desalted powder was dissolved in 10 mM LiPO_4_, pH 7.0 at a concentration of 2 mM. The stock solutions of both oligonucleotides were stored at 4°C. Solutions for fluorescence polarization studies were prepared at ∼1 µM concentration in 10 mM tetrabutyl ammonium phosphate, 1 mM EDTA, 200 mM KCl, pH 7.0 (referred to hereafter as tBAP folding buffer), denatured at 90°C in a water bath for 10–15 min, followed by annealing by slowly cooling to room temperature in the bath. Folding to the quadruplex state was checked by recording the CD spectrum of the sample over the wavelength range 340 nm to 220 nm and observing a maximum in the CD spectrum at 260 nm and a minimum at 240 nm.

**Table 1:** Oligodeoxynucleotides used in this study.

Name	Sequence	MW	ε_260 nm_ (mM^−1^ cm^−1^)
hTERT	5′-GGG GAG GGG CTG GGA GGG CCC GGA GGG GGC TGG GCC GGG GAC CCG GGA GGG GTC GGG ACG GGG CGG GG-3′	21632.8	659.3
1XAV	5′-TGA GGG TGG GTA GGG TGG GTA A-3′	6991.6	228.7

### Reagents

Thiazole orange, whose fluorescence quantum yield increases significantly when bound to DNA [25], was from Sigma Chemical Co. (St. Louis, MO). A stock solution of 250 µM dye was prepared in folding buffer containing 200 mM KCl and 20% sucrose. The final concentrations of the dye and oligonucleotide in the fluorescence polarization experiments was estimated by spectrophotometry with a Jasco V-550 instrument in conjunction with the appropriate absorption coefficient for the DNA along with a value of ε_500 nm_ of 63 mM^−1^ cm^−1^ for the dye. To ensure a negligible concentration of free dye, polarization experiments were carried out with excess DNA (DNA:dye≈3∶1).

KCl, tetrabutylammonium dihydrogen phosphate, tetrabutylammonium hydroxide and EDTA (acid form) were from Sigma. Sucrose was from Mallinckrodt Chemicals, Phillipsburg, NJ, and LiH_2_PO_4_ and LiOH monohydrate were from Aldrich Chemicals, Milwaukee, WI.

### Circular Dichroism and Thermal Denaturation

Thermal denaturation of quadruplexes was monitored using a Jasco J-810 spectropolarimeter (Jasco Inc., Easton, MD) equipped with a programmable Peltier thermostatted cell holder and a magnetic stirrer. CD spectra were collected using instrumental parameters: 280<λ<350 nm, 1.0 nm step size, 200 nm/min scan rate, 1.0 nm bandwidth, 2 s integration time, with 4 total scans averaged. For melting experiments, samples at 3–4 µM in a 1-cm path length cuvette were equilibrated in the cuvette holder at 4°C prior to starting the melt. Melting experiments were carried out with the thermal parameters: 4°C/min ramp, 0.05°C equilibration with a 60 s delay prior to acquisition. Spectra were corrected by subtracting a solvent blank. Melts were carried out in duplicate on successive days; data presented here are from the second melt. CD data were normalized to molar circular dichroism (Δε) based on DNA strand concentration using equation (1)
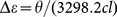
(1)where θ is the CD ellipticity in millidegrees, c is DNA concentration in mol/L, and l is the path length in cm.

### Analytical Ultracentrifugation

Sedimentation velocity measurements were carried out in a Beckman Coulter ProteomeLab XL-A analytical ultracentrifuge (Beckman Coulter Inc., Brea, CA) at 20.0°C and at 50,000 rpm in standard 2 sector cells. Data (200 scans collected over a 10 hour centrifugation period) were analyzed using the program Sedfit in the continuous c(s) mode or by a model assuming discrete, noninteracting species (www.analyticalultracentrifugation.com). Buffer density was determined on a Mettler/Paar Calculating Density Meter DMA 55A at 20.0°C and buffer viscosity was measured on an Anton Paar Automated Microviscometer AMVn. For the calculation of frictional ratio, 0.55 mL/g was used for partial specific volume and 0.3 g/g was assumed for the amount of water bound. hTERT sequences were dissolved to give a final concentration of 1 mM in tBAP folding buffer, diluted to give an absorbance at 260 nm of 0.5, heated in a boiling water bath for 10 minutes and allowed to cool to room temperature before centrifugation.

### Molecular Dynamics Simulations and HYDROPRO Calculations

Molecular models of G-quadruplex structures were created using the parallel quadruplex structure 1XAV from the Protein Data Bank with manual modification of the loop regions to for the hTERT sequence. Appropriate coordinating ions were added to the stacked G-tetrads of each model and additional ions were added to neutralize the G-quadruplex structures. The system was solvated in a rectilinear box of TIP3P water molecules with 15 Å buffer. The system was equilibrated using the following protocol: (i) minimize water and ions (1000 steps - 500 steepest descents) holding the DNA fixed (50 kcal/mol/Å), (ii) 50ps MD (heating to 300 K) with 20 ns MD as the production trajectory. A further 10 ns of accelerated MD production trajectory was obtained [26]. Simulations were performed in the isothermal isobaric ensemble (P = 1atm, T = 300K) using sander and GPU version of pmemd (AMBER 13). Periodic boundary conditions and Particle-Mesh-Ewald algorithms were used. A 2.0 fs time step was used with bonds involving hydrogen atoms frozen using SHAKE. Analysis of the trajectory was performed using the *cpptraj* module of the AmberTools 13 Package. Calculations of hydrodynamic properties were done using the program HYDROPRO 10 [27] using the recommended quadruplex parameters [28] on 5000 snapshots of the accelerated MD trajectory.

### Fluorescence experiments

Fluorescence excitation, emission, and polarization spectra were determined with a Jasco FP-6500 fluorescence spectrophotometer equipped with an ADP-303T Peltier temperature controller and an APH-103 fluorescence polarization unit (Jasco, Inc., Easton, MD). Instrumental settings were: λex = 510 nm, λem = 530 nm, 5 nm emission and excitation bandwidth, 2 s response time. Excitation and emission spectra were corrected by subtraction of a solvent blank.

### Determination of rotational relaxation time

The rotational relaxation time of a particle is defined as the time required for it to rotate through an angle *θ* of 68.4° (*cosθ = 1/e*). This time depends on the volume *V* of the molecule as well as the viscosity *η* and temperature *T* of the medium through the relationship *ρ = 3ηV/RT*. The rotational relaxation time *ρ_0_* for a spherical molecule without bound solvent can be calculated from the relationship *ρ_0_ = 3ηM* 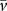 */RT*, where *M* is the molecular weight and 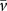 is the partial specific volume (taken as 0.55 cm^3^/mol for DNA quadruplexes). The ratio *ρ/ρ_0_* is considered to indicate deviations from a spherical shape and/or hydration of the molecule. The rotational relaxation time as defined above is related to the rotational correlation time *φ* (the time required for a molecule to rotate through 1 radian) by the equation *φ = 3 ρ* [29].

The rotational relaxation time of a fluorescently labelled molecule can be determined by measuring the degree of fluorescence polarization as a function of viscosity of the solution which can be varied by changing the temperature. Rotational relaxation times for the complexes of hTERT-FL and 1XAV with thiazole orange were determined in tBAP folding buffer with 200 mM KCl and 20% (w/v) sucrose at 2°C intervals over the temperature range 5 to 39°C. The viscosity of the sucrose solution at the experimental temperatures were obtained by interpolation (where necessary) from standard tables^6^.

The data sets were analyzed graphically as described by Montanaro and Sperti [30] using the Perrin equation (Eq. 2) which relates the degree of fluorescence polarization *P* to the rotational relaxation time *ρ* of the fluorescent particle and *τ*, the lifetime of the excited state: 
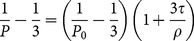
(2)
*P* is defined as *(I_║_−G⋅I_┴_)/(I_║_+G⋅I_┴_)*, where *I_┴_* is the emission intensity with the excitation polarizer at 90° (vertical orientation) and the emission polarizer is at 0° (horizontal orientation), and *I_║_* is the intensity with both polarizers at 90°. *G* is a grating correction factor* = i_┴_/i_║_* with *i_┴_* indicating the excitation polarizer is at 0° and the emission polarizer is at 90°, and *i_║_* indicating that both polarizers are in the 0° orientation. *P_0_* is the intrinsic polarization. The quantity *(1/P – 1/3)* is plotted vs. *T/η* and *ρ* is estimated from the slope and intercept estimated by linear regression.

### Determination of fluorescence lifetime

The fluorescence lifetime *τ* for the excited state of thiazole orange bound to hTERT and 1XAV was determined with an ISS K2 Multifrequency Phase Fluorometer (ISS, Champaign, IL). The sample was excited at room temperature with a 468-nm LED and polarizers set at “magic angle” conditions. Emission was measured through a 520-nm band pass filter (Newport Corp.). The instrument was calibrated with fluorescein in 0.1 M NaOH (lifetime = 4.0 ns). Phase and amplitude modulation data were analyzed with the ISS program Vinci Beta 1.7 (ISS) to determine lifetimes.

## Results and Discussion

**Figure 2:**
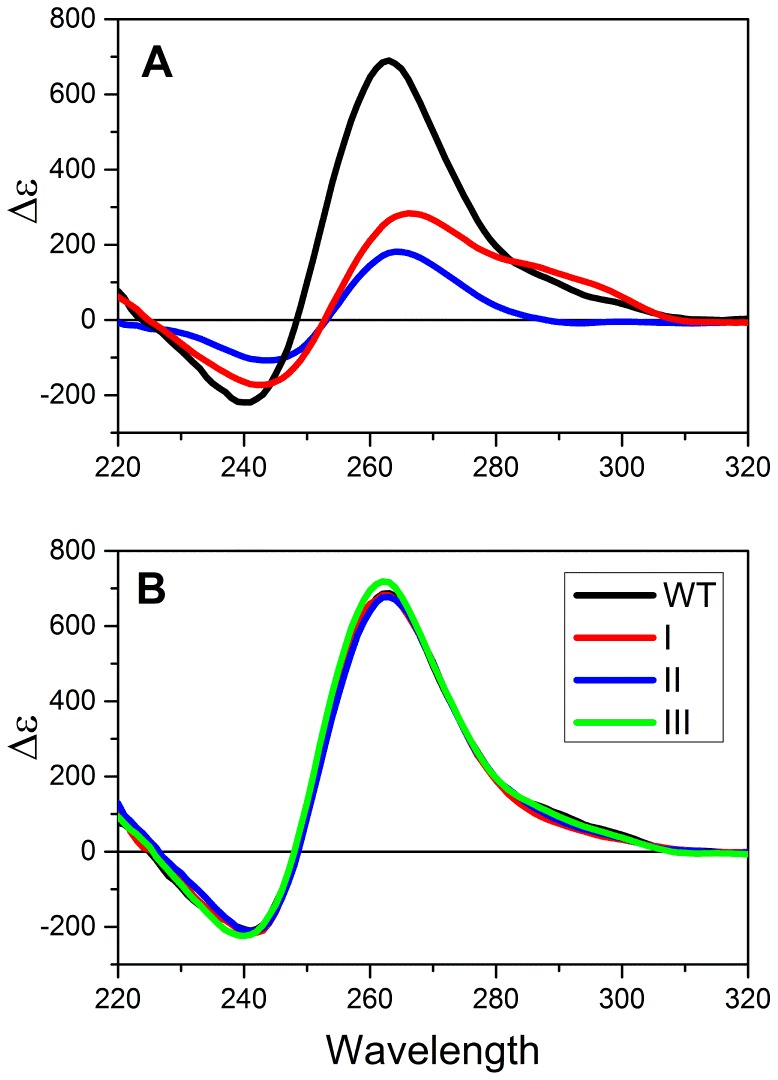
Circular dichroism studies of hTERT and mutant sequences. (A) Molar circular dichroism (Δε) of the hTERT promoter sequence in solution (black). The spectrum predicted for the structure shown in Fig. 1 is shown in red. The experimentally observed spectrum for the parallel quadruplex structure formed by the c-myc promoter sequence variant 1XAV is shown in blue. (B). Molar circular dichroism of the folded wild-type and mutant sequences shown in Fig. 1.

In order to characterize the effects of the observed mutations on the structure and stability of the hTERT core promoter, we initiated several biophysical studies. Fig. 2 shows circular dichroism (CD) spectra for the folded promoter and sequences containing the reported mutations. For the wild-type sequence, the observed CD spectrum is characteristic of a parallel quadruplex structure [31] and is notable for the exceptionally high amplitude of its molar circular dichroism. The observed spectrum is inconsistent with what would be expected for the proposed structure by Palumbo *et. al* [6] (Fig. 1). That structure predicts a spectrum that would be a linear combination of the spectra of a parallel quadruplex, an antiparallel hybrid quadruplex and an 8 bp duplex hairpin. We estimated the predicted spectrum by summing experimental molar circular dichroism spectra for 1XAV (Table 1), a human telomere hybrid quadruplex form and an 8 bp hairpin duplex. The predicted spectrum for that structure, shown in red in Fig. 2, differs significantly from the experimentally observed spectrum, especially in the amplitude at 260 nm. For comparison, the spectrum of a three-quartet parallel quadruplex formed by a sequence variant of the c-myc promoter sequence, 1XAV [15], is shown. The shape of that spectrum is similar to the observed hTERT spectrum but the amplitude at 260 nm differs dramatically. The difference in amplitudes can be quantitatively explained if the hTERT structure contains 9 stacked quartets, a structure that might result from the presence of three contiguous parallel quadruplexes that stacked upon one another. Such a structure is a reasonable alternative to the one shown in Fig. 1. Indeed, an hTERT promoter structure consisting of three quadruplexes was previous proposed based on CD spectroscopy and a polymerase stop assay [32] although an actual structure was not proposed.

**Figure 3:**
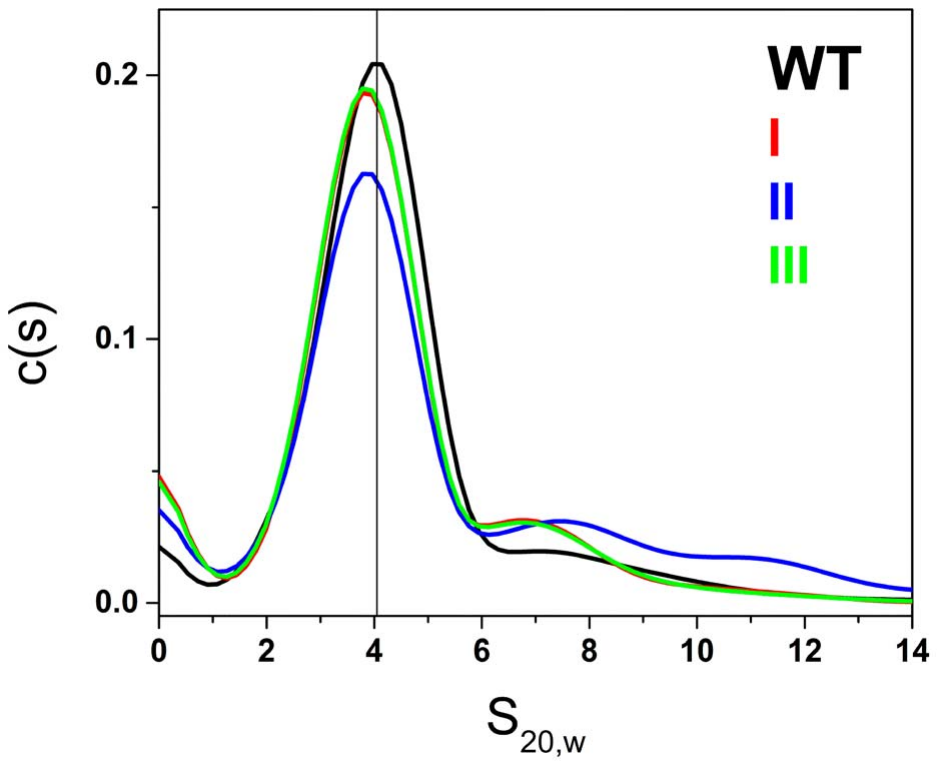
Results of sedimentation velocity experiments of hTERT promoter sequences in solution. The distribution of sedimentation coefficients, corrected for viscosity and temperature, are shown, revealing a major species with S_20,w_ of 4.05+/−0.04 for the folded wild-type sequence. S_20,w_ values for folded mutant sequences are slightly but significantly reduced to 3.5 - 3.6 S_20,w_.

Fig. 3 shows the results of characterization of the hTERT promoter by sedimentation velocity ultracentrifugation. The distribution (c(s)) of sedimentation coefficients is shown and reveals a major species along with a small amount of higher-order species. Analysis of these data using a model of discrete noninteracting species yielded an S_20,w_ value of 4.05±0.04 for the major (77%) component. The frictional ratio of this hydrated structure is 1.2, indicative of a nonspherical, asymmetric object [33]. The mass of this species corresponds to the molecular weight of a single strand of the sequence shown in Fig. 1, indicating a folded unimolecular structure. Steady-state fluorescence polarization experiments (S1–S2 Figs.) yielded a rotational relaxation time of 30.9±4.1 ns, compared to a predicted value of 14.6 ns for an equivalent sphere. The ratio of these two values, 2.1±0.3, again indicates that the folded unimolecular structure is asymmetric.

Minor amounts (14%) of faster sedimenting species (probably aggregates) are seen, along with minor amounts of slower sedimenting material (probably incompletely folded products). We previously determined that human telomere sequences can form structures with three contiguous quadruplexes with S_20,w_ values of 3.49 and 3.87 for antiparallel and all-parallel conformations, respectively [34]. These values suggest that the hTERT sequence forms a three quadruplex structure of some form, consistent with the conclusion based on CD spectra. A three-dimensional molecular model of mixed quadruplex-duplex structure shown is Fig.1, optimized with explicitly hydrated molecular dynamics, is predicted to have an S_20,w_ value of 3.2, significantly lower than experimentally observed value, again suggesting that the model is inconsistent with the observed behavior. For mutated sequences, discrete sedimentation coefficients are reduced to 3.5–3.6±0.04 S_20,w_ (Fig. 3). The differences in S_20,w_ between the wild-type and mutant sequences are significant (p<0.001) given the precision of sedimentation velocity measurements and as determined by a one-way analysis of variance of the experimental data. The reduced S_20,w_ values indicate hydrodynamically expanded structures compared to the wild-type sequence. Mutations thus seem to unfavorably affect packing of the multiple quadruplex structures. In addition to the reduction in S_20,w_ values, mutant sequences show a greater propensity to form aggregated structures, with a concomitant reduction (by 10–15%) in the amount of the major unimolecular species.

**Figure 4:**
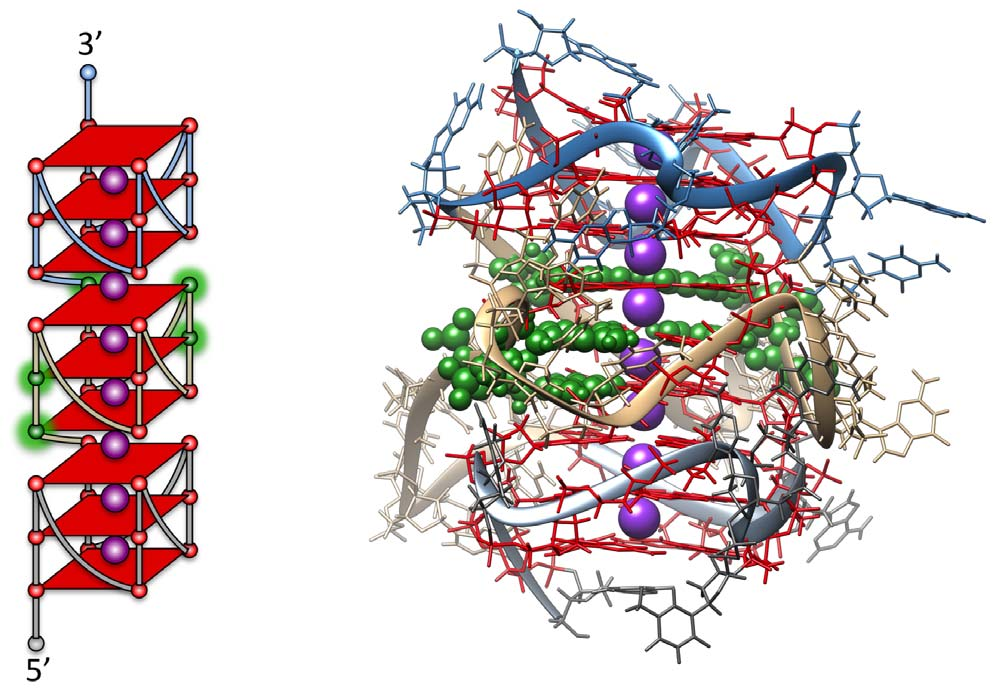
Molecular model for the hTERT promoter structure featuring three stacked parallel quadruplexes (right). The model predicts S_20,w_ = 4.00±0.1, in excellent agreement with the observed value of the wild-type sequence. The sites of reported mutated bases within the central quadruplex are colored in green. A schematic of the structure is shown on the left side, with the sites of mutations highlighted in green.

Molecular modeling simulations were used to construct a more realistic detailed model of the structure formed by the hTERT core promoter that is consistent with the biophysical data using computational protocols developed in our laboratory [28]. The three-stacked parallel quadruplex model (Fig. 4) was built using known structures with adjustment of the loop regions to be consistent with Micheli *et al* [32]. The 5′-region is the same as the reported NMR structure for a portion of the hTERT promoter sequence [35]. It is possible to construct a model that has all of the major mutations in the central quadruplex G-quartets. The model was fully stable while running a fully solvated 20 ns molecular dynamics trajectory with no disruption to quadruplex or inter-quadruplex stacking. This was followed by 10 ns of accelerated molecular dynamics to sample more conformational space of the loop regions. Hydropro calculations, using our recent quadruplex optimized calibration protocol [28], on 5,000 snapshots from the accelerate molecular dynamics trajectory revealed a range of S_20,w_ values of 3.95–4.03, in excellent agreement with what was experimentally observed. The resulting structures are (and have to be) extremely compact to maintain these sedimentation values. This structure is predicted to have a rotation relaxation time of 34.4 ns, in excellent agreement with the experimentally measured value. Several alternate models were explored, but none of these predicted hydrodynamic values that agreed with the experimentally measured values. For example, one alternate model was created that maintained the same three quadruplexes but did not have any inter-quadruplex stacking. The calculated S_20,w_ was 3.1 for this “beads-on-a-string” parallel structure, far from the experimental value and indicating that noninteracting quadruplex formation alone cannot account for the biophysical data. Similarly, a three-quadruplex structure with antiparallel quadruplex units could not account for the observed biophysical data.

Sedimentation and circular dichroism data are thus entirely consistent with a compact structure in which three parallel quadruplexes are tightly stacked on one another. The complex quadruplex-duplex structure (5) and the multiple “beads-on-a-string” quadruplex structure (25) are inconsistent with our biophysical data.

Surprisingly, the mutations shown in Fig. 1 have little effect on the thermal stability of the hTERT structure (S3A Fig.). Under the ionic conditions used, thermal denaturation is incomplete at 95°C and the melting temperature at the transition midpoint is greater than 90°C. However if the sequence 5′-GGGGCTGGGCCGGGGACCCGGG is used to form a monomeric quadruplex mutations dramatically decrease the thermal stability (S3B Fig.). That sequence would encompass the central quadruplex-forming sequence in the hTERT promoter and readily forms a single parallel quadruplex structure. In the full length hTERT structure allosteric interactions between contiguous quadruplexes apparently mask the destabilizing effects of the mutations. Additionally, we have shown that low-resolution techniques, such as melting, mask the complexity of quadruplex ensemble components [36].

While the mutations can be accommodated by alternate G-quadruplex formation in the longer sequences (which could account for the similar thermal stability), this would require a reduction to a two-tetrad stacked central quadruplex or by mixed guanine-adenine stacking. However, this would require changes in the connecting and internal loop structures of the central quadruplex, thus lowering the S_20,w_ value due to longer loops and decreased compactness, as is in fact observed in Fig. 3. Such alterations would affect recognition elements, protein binding and/or stability.

Detailed structural studies on quadruplex forming sequences in promoter regions have been limited to single quadruplex structures and often these shorter sequences are highly manipulated to reduce polymorphism. This study indicates that the longer promoter region QFS may be much more complicated and more biologically contextual. This may well be a general phenomenon as these extended QFS are common in the promoters of proto oncogenes that have not been previously examined in this detail.

More than 90% of human tumors overexpress telomerase, as do rapidly dividing cells such as stem cells and germ cells [37], [38]. The mechanism by which hTERT expression is dysregulated has been largely unknown. The data presented here indicate that the common mutations in the hTERT promoter occur in a quadruplex structure in this region. It is possible, by altering recognition elements and stability of this region, that the “transcriptionally active” duplex DNA structure with the ETS binding site would be favored. Importantly, ETS binding likely helps stabilize the double stranded (transcriptionally active) structure. This loss of quadruplex stability could abrogate the gene silencing effects of quadruplex formation, allowing increased hTERT expression. Because of its ubiquitous overexpression and its critical role in almost all tumors, telomerase is an excellent therapeutic target [39], [40]. The concept of reversing promoter silencing via mutations that cause quadruplex destabilization is an exciting new paradigm and provides the first plausible rationalization of mutation mediated gene transcription by quadruplex control.

## Supporting Information

S1 Fig **Representative response curves for phase delay and modulation ratio for determination of fluorescence lifetimes of thiazole orange bound to oligonucleotide 1XAV (panel A) and hTERT (panel B).** The points represent the experimental data and the lines represent the best fit of the data points using the lifetimes and fractional contributions of each to a two-lifetime model. The lower panels show the residuals for each fit. The data were analyzed using the program Vinci Beta 1.7. Experimental conditions: 0.9 µM 1XAV, 0.3 µM thiazole orange; 1.4 µM hTERT, 0.4 µM thiazole orange. Determinations were made at room temperature (∼21°C). Both samples were in tBAP folding buffer, 200 mM KCl, 20% sucrose, pH 7.0. Lifetimes and fractions determined by non-linear least squares analysis are shown in the figures.
(DOCX)

S2 Fig **Representative Perrin plots showing the dependence of the fluorescence polarization of oligonucleotide-bound thiazole orange on** ***T/η*** **.** Panel A shows data for 0.9 µM 1XAV and 0.3 µM thiazole orange. Panel B shows data for 1.4 µM hTERT with 0.4 µM thiazole orange. Experimental conditions were tBAP folding buffer, 200 mM KCl, 20% sucrose, pH 7.0. The temperature was varied from 5°C to 39°C in 2-°C intervals. For this particular set of experiments, *ρ* = 8.8 ns for 1XAV and 31 ns for hTert.
(DOCX)

S3 Fig **Thermal denaturation of (A) the long hTERT sequences shown in** **fig. 1** **and (B) truncated sequences encompassing the central quadruplex region.**
(DOCX)
