# Supplementary material for: An Improved Model for the hTERT Promoter Quadruplex
Source: PLoS One. 2014 Dec 19;9(12):e115580. doi: 10.1371/journal.pone.0115580 (PMC4272262; doi:10.1371/journal.pone.0115580)
Supplement: S1 Fig — Representative response curves for phase delay and modulation ratio for determination of fluorescence lifetimes of thiazole orange bound to oligonucleotide 1XAV (panel A) and hTERT (panel B). The points represent the experimental data and the lines represent the best fit of the data points using the lifetimes and fractional contributions of each to a two-lifetime model. The lower panels show the residuals for each fit. The data were analyzed using the program Vinci Beta 1.7. Experimental conditions: 0.9 µM 1XAV, 0.3 µM thiazole orange; 1.4 µM hTERT, 0.4 µM thiazole orange. Determinations were made at room temperature (∼21°C). Both samples were in tBAP folding buffer, 200 mM KCl, 20% sucrose, pH 7.0. Lifetimes and fractions determined by non-linear least squares analysis are shown in the figures. (DOCX) [file pone.0115580.s001.docx]

*Supporting Information for*

**An Improved Model for the hTERT Promoter Quadruplex**

Jonathan B. Chaires, John O. Trent, Robert D. Gray, William L. Dean, Robert Busgaglia, Shelia D. Thomas and Donald M. Miller

James Graham Brown Cancer Center, Department of Medicine, University of Louisville, Louisville, KY

| **A**  | **B**  |
| --- | --- |
| **Figure S1**. Representative response curves for phase delay and modulation ratio for determination of fluorescence lifetimes of thiazole orange bound to oligonucleotide 1XAV (panel A) and hTERT (panel B). The points represent the experimental data and the lines represent the best fit of the data points using the lifetimes and fractional contributions of each to a two-lifetime model. The lower panels show the residuals for each fit. The data were analyzed using the program Vinci Beta 1.7. Experimental conditions: 0.9 µM 1XAV, 0.3 µM thiazole orange; 1.4 µM hTERT, 0.4 µM thiazole orange. Determinations were made at room temperature (~21 °C). Both samples were in tBAP folding buffer, 200 mM KCl, 20% sucrose, pH 7.0. Lifetimes and fractions determined by non-linear least squares analysis are shown in the figures. | |
