# Supplementary material for: An Improved Model for the hTERT Promoter Quadruplex
Source: PLoS One. 2014 Dec 19;9(12):e115580. doi: 10.1371/journal.pone.0115580 (PMC4272262; doi:10.1371/journal.pone.0115580)
Supplement: S2 Fig — Representative Perrin plots showing the dependence of the fluorescence polarization of oligonucleotide-bound thiazole orange on T/η . Panel A shows data for 0.9 µM 1XAV and 0.3 µM thiazole orange. Panel B shows data for 1.4 µM hTERT with 0.4 µM thiazole orange. Experimental conditions were tBAP folding buffer, 200 mM KCl, 20% sucrose, pH 7.0. The temperature was varied from 5°C to 39°C in 2-°C intervals. For this particular set of experiments, ρ = 8.8 ns for 1XAV and 31 ns for hTert. (DOCX) [file pone.0115580.s002.docx]

*Supporting Information for*

**An Improved Model for the hTERT Promoter Quadruplex**

Jonathan B. Chaires, John O. Trent, Robert D. Gray, William L. Dean, Robert Busgaglia, Shelia D. Thomas and Donald M. Miller

James Graham Brown Cancer Center, Department of Medicine, University of Louisville, Louisville, KY

| **A**   | **B**   |
| --- | --- |
| **Figure S2**. Representative Perrin plots showing the dependence of the fluorescence polarization of oligonucleotide-bound thiazole orange on *T/η.* Panel A shows data for 0.9 µM 1XAV and 0.3 µM thiazole orange. Panel B shows data for 1.4 µM hTERT with 0.4 µM thiazole orange. Experimental conditions were tBAP folding buffer, 200 mM KCl, 20% sucrose, pH 7.0. The temperature was varied from 5 °C to 39 °C in 2-°C intervals. For this particular set of experiments, *ρ* = 8.8 ns for 1XAV and 31 ns for hTert. | |
