# Supplementary material for: An Improved Model for the hTERT Promoter Quadruplex
Source: PLoS One. 2014 Dec 19;9(12):e115580. doi: 10.1371/journal.pone.0115580 (PMC4272262; doi:10.1371/journal.pone.0115580)
Supplement: S3 Fig — Thermal denaturation of (A) the long hTERT sequences shown in fig. 1 and (B) truncated sequences encompassing the central quadruplex region. (DOCX) [file pone.0115580.s003.docx]

*Supporting Information for*

**An Improved Model for the hTERT Promoter Quadruplex**

Jonathan B. Chaires, John O. Trent, Robert D. Gray, William L. Dean, Robert Busgaglia, Shelia D. Thomas and Donald M. Miller

James Graham Brown Cancer Center, Department of Medicine, University of Louisville, Louisville, KY

**Figure S3**. Thermal denaturation of (A) the long hTERT sequences shown in figure 1 and (B) truncated sequences encompassing the central quadruplex region.

**
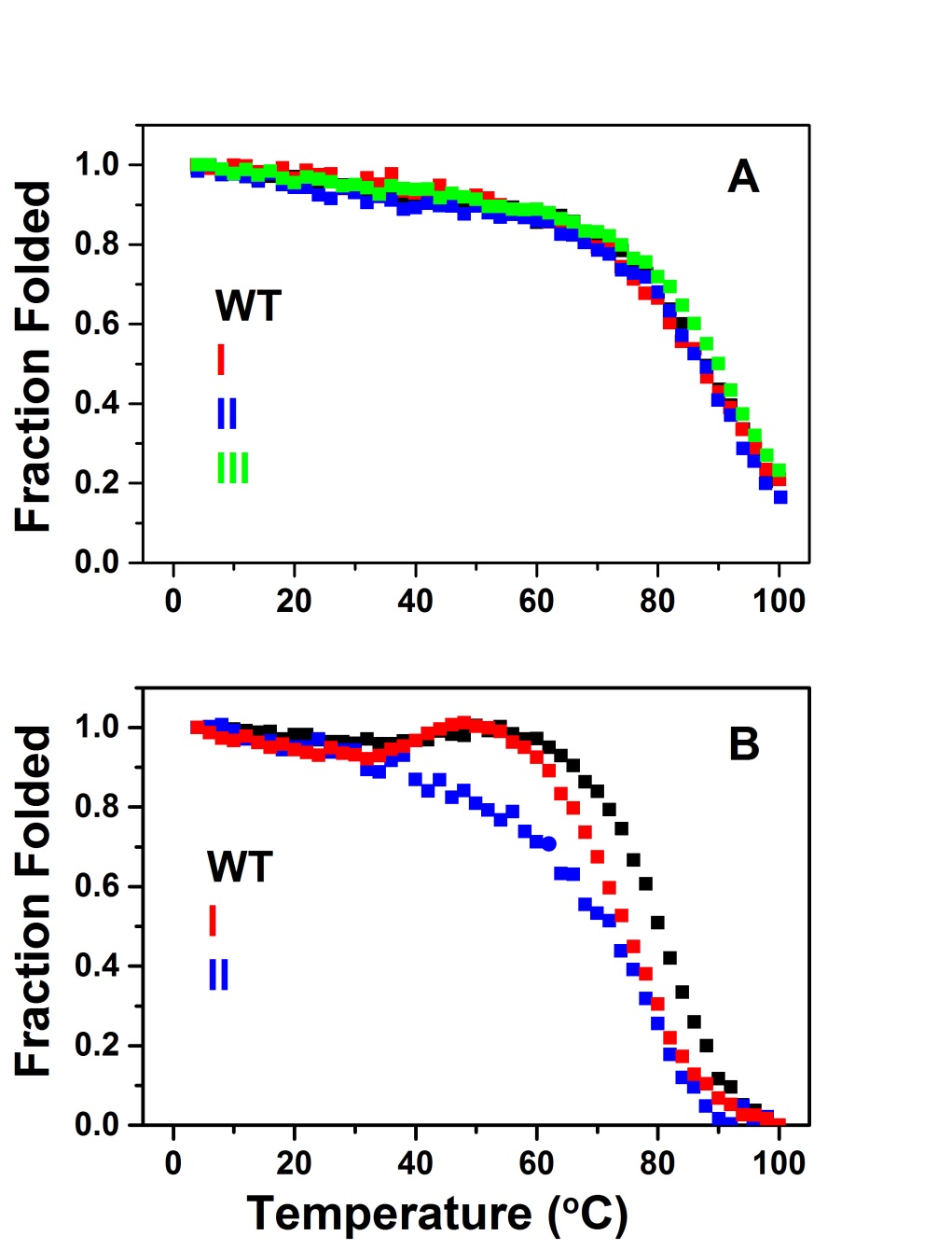
**
